# Claudins Overexpression in Ovarian Cancer: Potential Targets for Clostridium Perfringens Enterotoxin (CPE) Based Diagnosis and Therapy

**DOI:** 10.3390/ijms140510412

**Published:** 2013-05-17

**Authors:** Diana P. English, Alessandro D. Santin

**Affiliations:** Department of Obstetrics, Gynecology and Reproductive Sciences, Yale University School of Medicine, New Haven, CT 06520, USA; E-Mail: diana.english@yale.edu

**Keywords:** tight junction, claudin, clostridium perfringens enterotoxin, ovarian cancer, immuno-therapy

## Abstract

Claudins are a family of tight junction proteins regulating paracellular permeability and cell polarity with different patterns of expression in benign and malignant human tissues. There are approximately 27 members of the claudin family identified to date with varying cell and tissue-specific expression. Claudins-3, -4 and -7 represent the most highly differentially expressed claudins in ovarian cancer. While their exact role in ovarian tumors is still being elucidated, these proteins are thought to be critical for ovarian cancer cell invasion/dissemination and resistance to chemotherapy. Claudin-3 and claudin-4 are the natural receptors for the *Clostridium perfringens* enterotoxin (CPE), a potent cytolytic toxin. These surface proteins may therefore represent attractive targets for the detection and treatment of chemotherapy-resistant ovarian cancer and other aggressive solid tumors overexpressing claudin-3 and -4 using CPE-based theranostic agents.

## 1. Introduction

Claudins are a family of tight junctional proteins which are highly expressed in both benign and malignant ovarian tumors [1]. Normal epithelial cells are held together by tight junctions (TJs), adherens junctions (AJs), and gap junctions. TJs are the apical cell-cell adhesions that are important for epithelial cell polarity and regulate paracellular permeability by blocking the free diffusion of proteins and lipids between the apical and baso-lateral domains of the plasma membrane [2,3]. TJs are comprised of multiple membrane proteins such as occludin and claudin family proteins and several other associated peripheral proteins such as zonula occludens 1-3 (ZO-1, -2 and -3) [3,4]. These proteins are seen at the cell membrane interface where they contribute to the formation of the TJ and interact to form the diffusion barrier. Epithelial TJs are considered to be dynamic structures and the correlation of epithelial breakdown or dysfunction with the promotion of the neoplastic process has been suggested by previous studies [5].

Claudins have been shown to be essential and sufficient to form TJ strands and account for some of the selective variability of different barriers [6,7]. There is evidence that disruption of the cell to cell adhesion is a critical step in the process of cellular transformation and tumor cell metastasis [8]. The role of the claudins in this process is continuously being explored with new discoveries still occurring. Apart from contributing to mechanical cell adhesion at epithelial and endothelial cell interfaces, claudins also have the capacity to recruit cell signaling proteins and as such may regulate cell proliferation, differentiation and subsequent neoplastic transformation [9,10].

There are 27 different types of claudins identified to date with varying cell- and tissue-specific expression [11]. The expressions of claudins may also vary in different parts of the same organ. Most tissues express multiple claudins. The different claudin members may interact within a given tissue and this combination of the claudin proteins is thought to determine the strength and selectivity of the TJs. As they are cell surface proteins, most claudin positive tumor cells will show strong cell membrane staining with weak if any cytoplasmic reactivity noted in these cells. Of interest, the deregulation of the mitogen-activated protein kinase pathway can lead to the mis-localization of TJ proteins, including the claudins [12]. The delocalization of claudin proteins from cell membranes is common among transformed cells and in ovarian cancer this is associated with tumor cell migration and invasion [10,13]. Normal ovarian surface epithelial cells do not express either claudin-3 or claudin-4, however these claudins are both expressed at high levels in the majority of ovarian cancers [14–26].

Claudin-3 and -4 function as receptors for *Clostridium perfringens* enterotoxin (CPE), a potent cytolytic toxin. The use of this enterotoxin may be therefore exploited for therapeutic and diagnostic benefit for claudin-3 and -4 expressing tumors. Claudin expression has been identified in other gynecologic tumors as well, including cervical (preneoplastic and neoplastic lesions) and endometrial adenocarcinomas [27]. Some claudins also have been shown to have a prognostic role in particular tumor types, for example, claudin-3/-4 has a prognostic role in ovarian cancer, claudin-1 in colon cancer, claudin-10 in hepatocellular carcinoma and claudin-18 in gastric cancer [28–31].

## 2. Structure and Function of Claudins

**Figure 1:**
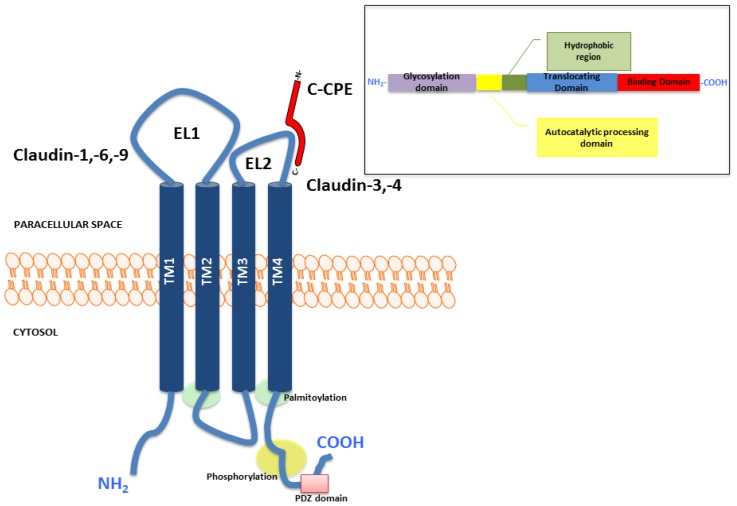
Representative structure of the claudin protein and the functional domains of *Clostridium perfringens* enterotoxin (CPE).

Claudins are known as tetraspan membrane proteins consisting of intracellular amino and carboxy terminals, 4 transmembrane domains and 2 extra-cellular loops mediating interactions between claudins on adjacent cells [2,32,33] (Figure 1). The amino acid sequences of the first and fourth transmembrane domains are highly conserved among the different claudin isoforms, however the sequences of the second and third domains are typically more diverse [34]. The second extracellular loop acts a binding site for *Clostridium perfringens* enterotoxin (CPE) in claudin-3 and -4 [35]. Claudin-3 and -4 consists of 220 and 209 amino acids respectively. Claudin-3 and -4 are considered to be the low and high affinity receptors for CPE respectively. Occludin, tricellulin and marvelD3 are other tetraspan transmembrane TJ proteins [4,36,37]. The scaffolding proteins like ZO-1, -2 and -3 and also signaling proteins are associated with TJs by binding of their PDZ-domains to respective binding sites at the carboxy terminus of claudins [38]. These membrane associated proteins govern the assembly and disassembly of TJ [32,39]. The carboxy terminus of most claudins contain potential serine and/or threonine phosphorylation sites [33]. The barrier function of claudins may be modulated through phosphorylation of the serine/threonine phosphorylation sites at the carboxy tail by various kinases such as cyclic AMP-dependent protein kinase and WNK4 [40]. The carboxyterminal tail is the region that shows the most sequence and size heterogeneity among the claudin proteins [34].

Claudins form the backbone of the TJ and are overall a highly structurally-related family of proteins with claudin-16 and -23 being the most different and claudins-6 and -9 are the most similar followed closely by claudin -3 and -4 and claudin -1 and -7 [41]. Some claudin genes have been found to be closely linked in terms of their proximity in the human genome, (for example claudin-3 and -4) [34]. It is however uncertain whether this genome arrangement has a function in coordinate regulation of the TJ. Interestingly, claudin-3 and -4 have been documented to have coordinate expression in several normal and neoplastic tissues and the combination is commonly found elevated in a variety of cancers [10,41,42]. In general, the 27 claudin genes that have been identified are typically small and have few introns or lack introns altogether. Claudin-3 and-4 have been localized at position 7q11, both of these genes have only one mRNA transcript form. Claudin-1 also has only one transcript form and the gene *CLDN1* is located at position 3q28. *CLDN5* can be found at position 22q11 and has two variants of mRNA that may be produced after transcription. *CLDN10* has also two variants of RNA transcript but has a gene position of 13q31. The claudin proteins show a wide range of sequence similarity and the size of these proteins is approximately in the range of 205 to 305 amino acids [34].

The claudins can be functionally divided into barrier-forming claudins such as claudin-1,-3,-4,-5 and pore forming claudins such as claudin-2,-7,-10 and -16 [38]. In a subtype dependent manner, the expression of barrier-forming claudins decreases paracellular permeability of ions, solutes and proteins while the expression of pore-forming claudins generally increases paracellular permeability to ions. As a result, the tissue specific expression of the different claudin isoforms will determine the permeability properties of the TJs in that tissue [43]. Most cells express multiple different claudin isoforms and these isoforms have the ability to co-polymerize into heteropolymers by homophilic and heterophilic interactions. The various types of claudin co-polymers then work together to regulate junctional permeability and to impart strength and selectivity to the TJ [16,44].

Based on the amino-acid sequence, the claudins may also be separated into two subgroups namely classic and non-classic claudins. Classic claudins are claudin-1 through -10, -14, -15, -17, -19 and share higher homology among each other compared to non-classic claudins (claudin-11, -12, -13, -16, -18, -20 through -24) [38]. Classic claudins are also more likely to share a common helix-turn-helix structure of the extracellular loop 2 which is involved in paracellular tightening [45,46].

While there still remains much to be uncovered about the claudin structure multiple studies have shown that abnormalities in claudins may result in the disruption of TJ barrier function as well as alter paracellular permeability. These structural abnormalities are known to be associated with a number of pathologic processes such as pulmonary edema, diarrhea, inflammatory bowel disease and kidney disorders [47–50]. Additionally, germline mutations in these genes can lead to familial diseases such as the autosomal recessive form of non-syndromic sensorineural deafness which results from a defect in the claudin-14 gene [51]. As such, it is apparent that proper cell to cell and cell to extracellular matrix interactions are essential for continued normal tissue and organ functioning. Similarly, the proteins constituting TJs, such as the claudins, are quite likely to have a central role in tumorigenesis and also in tumor spread.

Claudins are found in cell adhesions and are thought to facilitate the communication of the extracellular environment to both intracellular signaling pathways and to the cytoskeleton. Tight junction disruption in premalignant tissues can increase the likelihood of progression to a frankly invasive tumor due to passage of large solutes across epithelial barriers allowing growth factors (usually in luminal fluids in epithelial tissues) to now bind to their growth factor receptors (usually on the baso-lateral surface facing interstitial fluid and the bloodstream) and this interaction may lead to continuous stimulation of premalignant cells [13]. Claudin expression may also affect the epithelial permeability to substances such as growth factors and also modulate the response of other tight junction proteins to various types of injury [52].

The pattern of expression of claudins in normal tissue, benign and malignant tumors is not only complex but also organ dependent [10,41,53]. Large scale serial analysis of the genome and gene expression arrays have documented higher expression of claudin-3, -4, -7 and -10 in ovarian carcinoma compared to normal ovarian surface epithelium [15,16,18,43,54]. These findings have been validated using quantitative reverse transcription-polymerase chain reaction (qRT-PCR) and immunohistochemistry (IHC). Generally, studies comparing the expression of claudins in benign or borderline ovarian tumors *versus* ovarian carcinoma have not been entirely conclusive [1,14,16,55,56]. However, most of the prevailing literature provides evidence that claudin-3 and -4 are highly differentially expressed in ovarian cancer and also correlate with chemo-resistance and poorer survival albeit some differing results have been reported depending on cell lines studied.

Claudin-1, -3, -4, -5 and -7 are the claudins most commonly overexpressed in ovarian tumors (Table 1). Overall, in reviewing several studies, it is evident that claudin expression is altered in a variety of tumors with the most commonly identified claudins to have an altered expression being claudin-1, -3, -4, -5, -7, -10 and -16 [10]. This phenomenon is likely due to the role of claudins in tumor survival and invasion, as it is not unusual for some carcinomatous tissue to lose their TJ proteins as they grow and develop [10,57–59], for example, claudin-1 and -7 are typically downregulated in hepatocellular carcinomas [5,60].

TJ-tight junction, HCV- hepatitis C virus, CPE- Clostridium perfringens enterotoxin, RPE- retinal pigment epithelium, EPCAM- epithelial cell adhesion molecule.

Claudin function is regulated at many sites including at the level of the tight junction as a result of crosstalk between tight junction components [64]. In addition several claudins are known to be phosphorylated by kinases which may affect both claudin position and function. Another potential mechanism of regulation of claudin expression is endocytic recycling of claudin proteins [65]. At the transcriptional level, there is evidence that transcription factors such as GATA-4 and Snail are able to bind to the promoter regions of several claudin genes and affect their expression [66,67]. The claudins may also be downregulated not only at the point of transcription but also post-transcriptionally via a variety of cytokines and growth factors [64,68].

Epidermal growth factor (EGFR) signaling has been demonstrated to modulate the expression of the claudins in various cell types [48,69,70]. Recently this mechanism of the TJ protein regulation in ovarian cancers was explored by treating both ovarian mucinous and serous cystadenocarcinoma cell lines with EGF [71]. EGF was found to downregulate claudin-3 in mucinous ovarian carcinoma cell lines and claudin-4 in ovarian serous cystadenocarcinoma by inducing the degradation of these proteins with also changes in the structure and function of TJ via the MEK/ERK or PI3K/AKT signaling pathway. The pretreatment with EGFR inhibitors, MEK/ERK inhibitors and PI3K/AKT inhibitors in the ovarian mucinous cystadenocarcinoma cell lines prevented the decrease of claudin-3 by EGF. On the other hand, for serous ovarian carcinoma cell lines, pretreatment with inhibitors of EGFR, MEK/ERK but not PI3K/AKT prevented the decrease in claudin-4 by EGF. This suggests alternative mechanisms for claudin regulation by EGF among the different ovarian carcinoma subtypes *in vitro*. These results provide evidence that EGF may affect claudin and TJ function in ovarian cancer cells during cancer development. Additionally, in ovarian serous cystadenocarcinoma cell lines, EGF was found to downregulate the cytotoxic effects of CPE via claudin-4. As a result, it is also theorized that EGF may affect effective claudin-4 targeting therapy with CPE in serous cystadenocarcinoma [71].

## 3. Claudins in Ovarian Cancer

### 3.1. Claudin-1 and Claudin-2

Claudin-1 expression has been studied and demonstrated in ovarian serous carcinoma and ovarian endometrioid carcinoma [72]. Claudin-1, like claudin-3 and -4, is an epithelial specific claudin protein. The expression of claudin-1 is elevated in many types of cancer cells and is proposed to be potentially causally involved in tumor growth and progression. Claudin-1 has been shown to have anti-apoptotic activity and is thought to play a role in the expression and localization of β-catenin and E-cadherin. As such claudin-1 plays a role in the epithelial to mesenchymal transition and the c-abl-Ras-Raf-1-ERK1/2 signaling axis is important in claudin-1 induced malignant progression [73,74].

Claudin-1 has been identified as one of the genes notably upregulated in ovarian cancer-initiating cells and claudin-1 overexpression in these cells leads to a low degree of cell differentiation and a high rate of invasive growth [75]. It has been discovered that microRNA-155 (miR-155) targets claudin-1 with specificity and the increased expression of endogenous mature miR-155 may have an inhibitory effect on human ovarian cancer-initiating cell proliferation and invasion *in vitro* and *in vivo* through its effect on limiting claudin-1 expression. Claudin-2 has not been noted as a tight junction protein with high expression in ovarian cancer. Data regarding the expression and function of claudin-2 mainly centers around hepatocellular, breast and gastrointestinal carcinomas as well as Paget’s disease [27,76,77].

### 3.2. Claudin-3 and Claudin-4

Epithelial ovarian carcinoma remains the gynecologic malignancy with the highest mortality rate [78]. Two-thirds of patients have advanced disease at the time of diagnosis and unfortunately the majority of patients will recur after an initial response to the combination of maximal cytoreductive surgery and combined platinum and paclitaxel-based chemotherapy [79,80]. Thus the identification of novel therapeutic approaches against chemotherapy resistant/recurrent ovarian cancer remains a high priority.

Ovarian cancers of varying subtypes including mucinous, serous, undifferentiated, clear cell, and endometrioid carcinomas have been found to highly express claudin-3 and claudin-4 but normal ovarian surface epithelium does not [14,59,81–83] (Table 2). This data suggests that the low-level expression of these claudins is associated with a benign condition and that high expression is more likely to be a signal of a malignant transformation. Consistent with this view the expression of claudin-3 and -4 in ovarian epithelial cells is thought to enhance neoplastic cell invasion and has been found to be associated with increased matrix metalloproteinase-2-activity and angiogenic effects [82]. Some research has also suggested that up-regulation of claudin-3 may be an early event in the development of epithelial ovarian cancer and have potential application in detection of early stage disease [56].

Using gene expression profiling, the differential patterns of expression between ovarian tumors and normal ovarian cells has been explored. Several groups including our own have recently used high throughput gene array technologies to compare the expression profiles of ovarian cancer to those of normal ovaries with the aim of identifying potential diagnostic and therapeutic markers for this aggressive malignancy. Claudin-3 and -4 genes have been reported to be highly differentially expressed in biologically aggressive malignancies including ovarian serous carcinoma (OSC) and the identification of claudin protein expression has proven to be of clinical relevance in this tumor and a variety of others [10,53]. The mechanism of the increased claudin-3 and-4 expression in ovarian carcinoma is thought to be the result of epigenetic modifications of the claudin promoter regions in the cancer cells resulting in increased cell survival, invasion and motility [59,93,94].

Our research group also examined the genetic fingerprints of ovarian serous cancer in flash-frozen tumor biopsies as well as primary and/or established ovarian cancer cell lines and compared the gene expression signature with that of normal cells including ovarian surface epithelium exposed to short-term culture or immortalized normal ovarian cell lines (HOSE). The gene expression in flash-frozen OSC was found to have a high correlation with those of purified primary ovarian serous carcinoma in short-term *in vitro* cultures. Claudin-3 and -4 were found among the most highly overexpressed genes in OSC compared to HOSE [15].

As the comprehensive study of the molecular signature of ovarian cancer has identified claudin-3 and -4 as top differentially expressed genes, next to be investigated was the gene expression profile in chemotherapy-naïve *versus* chemotherapy-resistant ovarian cancer. Chemotherapy-resistant ovarian cancer was found to express the claudin-3 and -4 genes at significantly higher levels when compared with chemotherapy-naïve ovarian tumors [95,96]. These ovarian cancer cell lines continued to display considerable sensitivity to CPE *in vitro* and *in vivo* regardless of their documented resistance to multiple chemotherapeutic agents [97].

There is also great interest in the mechanisms and markers of platinum-resistance secondary to the importance of this drug as first-line treatment of ovarian cancer whether in the neoadjuvant or adjuvant setting. However limited information is currently available about the exact mechanisms of cisplatin resistance in ovarian cancer including whether or not claudin-3 or -4 may play roles as influx or efflux transporters of cisplatin. It is postulated that claudin-3 or -4 overexpression may inhibit the penetration of chemotherapeutic agents into ovarian cancer tissue and as a result generate chemo-resistance [98].

Quantitative proteomic technology integrated with mRNA expression levels has been recently utilized in an effort to identify protein markers capable of prospectively determining chemo-resistant ovarian tumors [96]. In this study a total of 1117 proteins were identified and quantified in cisplatin-sensitive and -resistant ovarian cancer cells. The relative expression of 121 of these proteins varied between the cell lines with 58 of them found to be overexpressed in cisplatin-resistant cells. Claudin-4 was identified as one of the top proteins associated with cisplatin resistance in ovarian cells with a 7.2 fold overexpression level.

A Japanese research group reported similar results showing that claudin-4 expression was higher in ovarian cancer tissue from platinum-based chemo-resistant patients *versus* chemo-sensitive patients. In this study suppression of claudin-4 resulted in a significant increase of cisplatin sensitivity and cellular accumulation of fluorescence-labeled cisplatin. Claudin-4 expression was significantly greater in ovarian cancer tissue from chemo-resistant patients compared to chemo-sensitive patients. Thirty-three out of the 43 cases (76.7%) of patients with ovarian cancer examined had positive claudin-4 expression with a significant shorter survival noted in the claudin-4 positive *versus* claudin-4 negative group [98].

In contrast, a recent study by Shang *et al.* using two established cell lines provided some support to the notion that claudins-3 and -4 may serve to constrain the growth of human ovarian cancer xenograft and limit metastatic potential [42]. In this study knockdown of claudin-3 and -4 increased the *in vivo* growth rate and metastatic potential of the xenografted tumors and reduced expression of these claudin proteins enhanced cell migration and invasion in *in vitro* assays [42]. In the Shang *et al.* study, the loss of either claudin-3 or -4 resulted in the down-regulation of E-cadherin mRNA and protein as well as activation of β-catenin pathway signaling and as such claudin-3 and -4 may mediate interactions with other cells *in vivo* that result in reduced growth and metastatic potential through the maintenance of E-cadherin expression and by limiting β-catenin signaling [42]. E-cadherin is the major structural protein of the adherens junctions and loss of E-cadherin is declared as a hallmark of the epithelial-to-mesenchymal transition through which it is speculated that cells must pass before becoming metastatic [99,100]. It is known that E-cadherin acts as a negative regulator of the β-catenin signaling pathway, which is a pathway that guides cell destiny through the regulation of cell growth, motility and survival [42,101]. As such, down-regulation of E-cadherin as well as activation of β-catenin pathway signaling could account for the increased metastatic potential of the ovarian cancer cell lines studied. Of interest, low-level expression of claudin-3 and claudin-4 in other human solid tumors has also been linked to a mesenchymal pattern and, as such, correlates to an overall poor survival in breast, esophageal, colon and pancreatic carcinoma [102–105].

Importantly, the Shang *et al.* study also lends support to the body of evidence indicating that most ovarian cancers arise from the distal fallopian tube epithelium even though these cancers are largely accepted to arise from multiple locations including ovarian surface epithelium [106–108]. In this study, immunohistochemical analysis was performed for claudin-3 and -4 expression in both the distal fallopian tube and tumor in six cases of serous ovarian cancer. All six cases had high claudin-3 and -4 expression in both sites. As the majority of ovarian cancers show a high expression of these claudins, it has been postulated that ovarian cancer develops from an epithelium which at its baseline or preneoplastic state normally expresses these two proteins. This same group has recently demonstrated in a single cell line that knockdown of claudin-3 and -4 resulted in marked changes in the phenotype of ovarian cells including an increased resistance to cisplatin by regulating the expression of the copper influx transporter CTR1 [109].

Taken together the results of these latter studies are consistent with the conclusion that the effect of claudin-3/-4 knockdown on cisplatin resistance may be the consequence of promoting an epithelial to mesenchymal transition after the downregulation of the claudin proteins. This interpretation is supported by previous studies in gynecologic carcinosarcoma showing that high expression of claudin-3/-4 is present in the epithelial but not in the sarcomatous component of multiple carcinosarcomas studied by immunohistochemistry [89].

The differences in claudin-3/-4 expression by ovarian cancer subtype and the correlation with outcome in ovarian cancer patients has also been researched by several groups [1,14,55,110]. In one study, low claudin-3 protein expression was associated with a trend towards a poor survival in 115 primary ovarian carcinomas with 68.6% being of serous histology [56].

One large study found that claudin-4 was expressed in nearly 70% of the ovarian cancer tissues examined and was differentially expressed across ovarian cancer subtypes, with the lowest expression noted in clear cell ovarian carcinomas. The highest percentage of expression was detected in endometrioid and mucinous subtypes (both 77.4% positive) compared to serous (72.17%) and clear cell (57.58%) subtypes. Also no association was found between claudin-4 expression and disease-specific survival in any subtype [81]. In yet another study, claudin-3 and -4 were significantly up-regulated by 5-fold or more in most subtypes of ovarian epithelial carcinoma. By immunohistochemistry (IHC) claudin-3 was expressed in 81% and claudin-4 expressed in 85.7% of 84 serous adenocarcinomas respectively. Borderline serous tumors and adenomas had significantly lower expression of these proteins than the adenocarcinomas. The survival analysis in this study revealed that serous adenocarcinoma patients with high claudin-3 expression had a substantially shorter survival and multivariate analysis showed claudin-3 overexpression to be an independent negative prognostic factor [111]. Consistent with these results claudin-3 gene silencing with small interfering RNA has been shown in mouse models to suppress ovarian tumor growth and metastasis [112].

In contrast to these results Litkouhi *et al.* found the highest percentage of claudin-4 expression in clear cell and endometrioid subtypes of ovarian cancer however this study had a much smaller sample size which may at least partially explain the differing results. Also in this study, there was no statistically significant difference in survival found between the claudin-4 positive and claudin-4 negative groups [110].

Support for the role of claudins in promoting tumor progression has also come from studies evaluating the anatomic-site related expression and the prognostic role of claudins in ovarian cancer. In one particular study, the data of immuno-stains for claudin-1, -3, -4 and -7 on pleural effusions, corresponding primary tumors and solid metastasis of ovarian cancer were all gathered in order to identify associations between anatomic site, clinic-pathologic parameters and survival. It was found that all 4 claudins were expressed in >85% of tumors at all anatomic sites [1]. Moreover, with the exception of claudin-4, all the other claudins were upregulated in ovarian cancer effusions compared with solid tumors and that the expression of claudins-3 and -7 in pleural effusions independently predicts poor survival in ovarian cancer [28].

Facchetti *et al.* investigated the usefulness of claudin-4 in the diagnosis of mesothelioma and other malignancies that may mimic mesothelioma. In this study, analysis was performed on 454 tumors, including 82 mesotheliomas, 336 carcinomas of different origins, 36 non-epithelial spindle cell neoplasms as well as 97 cytological samples from a combination of reactive effusions, mesothelioma and metastatic carcinomas. Claudin-4 was consistently negative in normal and reactive mesothelium as well as in all 82 mesotheliomas but strong reactivity (using anti-claudin-4 primary antibody) was found in the significant majority of serosal metastasis from primary carcinomas particularly lung, breast, gastrointestinal tract, pancreas, ovary and primary peritoneal carcinoma. In effusions, metastatic tumor cells stained positive in 96.8% of cases. Facchetti’s study therefore suggested that claudin-4 may be a pan-carcinoma marker with high sensitivity and specificity and that this claudin protein may be considered a primary immunohistochemical marker to rule out the diagnosis of mesothelioma in patients with pleural and peritoneal biopsies and effusions [113].

### 3.3. Claudin-5

Claudin-5 is mainly present in vascular endothelial cells but is also seen in ovarian epithelial tumors but at a much lower frequency than claudins-1, -4 and -7 [1]. In a study of 60 different types of ovarian lesions, sex-cord stromal tumors and cysts were mainly negative for claudins-1, -4, -5, and -7. In immature teratomas, mostly the epithelial component was usually positive and the other components were negative. Dysgerminomas did not express any of the claudins-1, -4, -5, and -7. The authors findings in this study were that claudins-1, -4, and -7 were mainly expressed in epithelial ovarian tumors [14].

The role of claudin-5 and vascular endothelial growth factor (VEGF) in the development of malignant ascites was explored recently. Claudin-5 has been shown in an *in vitro* corpus luteum model to be important for the regulation of vascular permeability [114]. VEGF is produced by malignant cells including ovarian cancer cells and induces angiogenesis to promote tumor growth and survival. VEGF has also has been shown to enhance vascular permeability and influence endothelial TJs [115–117]. Up to 24-fold higher VEGF levels may be induced in malignant tumors compared to benign ovarian cysts [118]. These high VEGF levels are theorized to increase local permeability and result in fluid extravasation (third spacing) and thus ascites formation. The role of VEGF-dependent production of claudin-5 as a regulator of vascular permeability in ovarian cancer patients was thus investigated by studying the amount claudin-5 in peritoneal tissue as well as VEGF in serum and ascites. The researchers also established a co-culture system of both ovarian cancer cells and endothelial cells to examine whether a functional association exists between claudin-5 and increased peritoneal permeability.

The results showed that the serum and ascites of preoperative ovarian cancer patients had increased levels of VEGF and that there was a VEGF-dependent decrease of claudin-5 in endothelial cells co-cultured with ovarian cancer cells. The ovarian cancer patients had a lower amount of claudin-5 detected in the peritoneal vessels compared to healthy controls. The results suggest that one mechanism by which VEGF may induce ascites formation in ovarian cancer patients is by increasing peritoneal permeability secondary to the downregulation of the TJ protein claudin-5 in the peritoneal endothelium [119].

Interest in the expression of claudin-5 and its correlation with ovarian cancer behavior also arose. This was investigated in a Finnish study of 85 serous ovarian cancer tissue samples. There was an association between claudin-5 expression and cancer grade and stage. The highest claudin-5 expression was seen in patients with high grade and advanced staged disease. Cancer-specific and overall survival was also associated with claudin-5 expression. Only 25%–30% of claudin-5 positive patients were alive at 5 years follow-up compared to 60% of claudin-5 negative patients. This study therefore suggests that increased claudin-5 expression is associated with aggressive behavior in serous ovarian adenocarcinoma [120].

### 3.4. Claudin-6

Unlike claudin-3 and -4, which are expressed in multiple epithelial tissues, the expression of claudin-6 is more restricted and believed to be predominately found in embryonic tissues and in undifferentiated pluripotent stem cells [41,121]. In this regard, previous studies have reported that claudin-6 has an important role in the development of the mouse embryonic epithelium and endodermal tissues [122,123]. However, claudin-6 expression has been reported in multiple human cancers such as rhabdoid tumors, breast cancers and gastric cancers [124–126]. Importantly, our group has recently found that claudin-6 can be expressed in ovarian cancer and may represent a novel functional receptor for CPE [127]. Consistent with this view, UCI-101, an ovarian cancer cell line highly sensitive to CPE, does not express claudin-3/4 and knockdown of claudin-6 in these cells decreases CPE sensitivity. Moreover, different ovarian cell lines that are resistant to the effects of CPE can be made sensitive through claudin-6 overexpression. Finally, binding assays show that CPE can indeed bind claudin-6 in cells and that this binding is associated with CPE cytotoxicity. These results establish claudin-6 as a novel receptor for CPE and introduce the possibility of a novel therapeutic target for ovarian and other cancers that express claudin-6.

### 3.5. Claudin-7

Previous studies have shown that claudin-7 is up-regulated in endometriosis associated endometrioid ovarian cancer [84] and also frequently upregulated in other epithelial ovarian cancers along with claudins-3 and -4 [18,85,128]. High claudin-7 expression has been associated with a poor response to platinum-based chemotherapy in epithelial ovarian cancer [129]. However, claudin-7 is downregulated in several other cancers including head and neck, esophageal and prostate cancer [130–132]. In breast cancer, claudin-7 expression was not only found to be decreased but also to be inversely correlated with tumor grade and metastatic disease [57,133]. The exact reason for the differing pattern of expression in various cancers is largely unknown but is likely related to the specific role of this claudin in these malignancies. Dahiya *et al.* evaluated claudin-7 expression levels in 95 ovarian tissue samples and cell lines using western blotting, qRT-PCR analysis and IHC. The gene for claudin-7 was found to be upregulated in all tumor samples studied and small-interfering RNA-mediated knockdown of claudin-7 in ovarian cancer cells led to significant changes in the expression of other genes as determined by microarrays. Analyses of the genes differentially expressed revealed that the genes altered in response to claudin-7 knockdown were associated with pathways implicated in various molecular and cellular functions including cell cycle growth and proliferation, cell death and development. Claudin-7 expression was associated with a net increase in invasion but also a decrease in cellular migration. Claudin-7 was found to be universal upregulated in the most common epithelial ovarian cancer subtypes (serous, clear-cell, endometrioid and mucinous) at both the mRNA and protein levels. With the use of immunobloting and qRT-PCR, the authors demonstrated that mRNA levels and protein levels were not always correlated, suggesting post-translational regulation of claudin-7 in epithelial ovarian cancer. Overall this work shows that claudin-7 is significantly upregulated in epithelial ovarian cancer and may be functionally involved in ovarian carcinoma invasion, as such claudin-7 may also represent a potential marker for ovarian cancer detection and also a target for therapy [86].

The prognostic significance of claudin-7 overexpression in epithelial ovarian cancer patients including sensitivity to platinum-based chemotherapy was investigated in another study. In this study claudin-7 was found to be expressed in 69/71 (97.1%) epithelial ovarian cancers but not in normal ovaries (*p <* 0.001) and high claudin-7 expression in primary tumors correlated with shorter progression-free survival (PFS) of patients and poor sensitivity to platinum based chemotherapy. As such claudin-7 expression may also represent an independent prognostic factor for PFS and be important in regulating epithelial ovarian cancer response to platinum-based chemotherapy [129].

## 4. Claudin-3 and -4 are Potential Targets for CPE-Based Theranostics

The identification of tumor origin, the prediction of chemotherapy response and the determination of prognosis is not without merit but of even greater importance is the potential for the use of claudin-isoform specific targeting agents in malignancies with increased claudin protein expression. *Clostridium Perfringens enterotoxin* (CPE) has already been demonstrated to induce necrosis in xenograft models of claudin-4 expressing tumors [50,52,97]. There is however concern that the expression of claudin-4 on normal epithelia will limit the usefulness of this anti-tumor strategy. As ovarian carcinoma is largely a disease of the peritoneal cavity, the utilization of intra-peritoneal (i.p) treatment with full length CPE holds promise for this claudin-4 expressing tumor. Further strategies *in vivo* to limit CPE toxicity to normal tissues also expressing these proteins may include local delivery of the blocking CPE peptide fragment to gut and lung via enteral and inhalation routes respectively.

As surface proteins highly expressed in chemotherapy-resistant ovarian cancer, the claudins represent attractive therapeutic targets. Claudins-3 and -4 have been shown to represent the natural receptors for CPE and as such to be the main family members of the trans-membrane tissue-specific claudin proteins capable of mediating CPE binding and cytolysis [134]. Several strategies involving the use of CPE as a novel therapeutic and possibly even diagnostic compound have been investigated with more work underway in this area. In fact, multiple research groups have reported using claudin-4 as not only a therapeutic target for toxin delivery but also as a target for fluorescent molecules to assist with the localization of ovarian and breast cancer cells [98,135–138]. The binding of the CPE toxin to cells results in the formation of membrane pore complexes and rapid cell death. The clinical role of

CPE-targeted therapy therefore holds promise in claudin-3 and -4 expressing malignancy and more so has potential for the treatment of chemotherapy resistant disease [57,134,139–141]. Supporting this view, the functional cytotoxicity of CPE in metastatic androgen-independent prostate cancer overexpressing claudin-3 has been reported previously [140]. CPE is produced by the anaerobic gram-positive bacterium, *Clostridium perfringens* type A strain. This strain is known to cause food poisoning and is the second most commonly reported food-borne illness in the United States. CPE is a single polypeptide of 35 kDa composed of 319 amino acids [142]. The carboxy (*C*)-terminus of CPE allows for the binding, while the *N*-terminus of CPE is associated with cytotoxicity [128,143,144] (Figure 1). CPE triggers lysis of epithelial cells through interaction with the claudin-3 and claudin-4 receptors with resultant collapse of the cellular colloid-osmotic equilibrium and initiation of massive permeability changes leading to osmotic cell ballooning and lysis [134,142]. Not surprisingly, mammalian cells that do not express either claudin-3 or claudin-4 fail to bind CPE and are not susceptible to CPE cytotoxicity [143,145].

Although CPE is a recognized as a potential therapeutic agent, several new and promising agents cannot be utilized clinically due to undesirable pharmacokinetics and/or systemic toxicity. For a drug to be effective it must be able to cross the necessary tissue barriers in order to reach to its target without significant effect on normal tissues. As most TJ modulators previously were rendered less effective as a result of general low tissue specificity and side effects such as cell exfoliation due to epithelial cell barrier dysfunction, the *C*-terminal region of CPE emerged as a promising tool to modulate TJs in a tissue-specific and direct manner [146,147]. The side effects are expected to be less due to a more specific modulation of an important component of the TJ [147] and the activity is restricted to tissues that express the CPE- sensitive claudin-3 and -4. The *C*-terminal fragment of CPE (*C*-CPE peptide) has been shown to act to increase drug absorption through mucosal surfaces in a reversible and concentration-dependent manner. The *C*-CPE is also able to sensitize epithelial ovarian cancer cells to the cytotoxic effects of Taxol and Carboplatin at relatively low doses in a claudin-4 dependent manner. Also compared with single agent Taxol or Carboplatin, the addition of *C*-CPE to Taxol is able to significantly suppress large tumor burdens in animals via inhibiting tumor cell proliferation and accelerating apoptosis [148]. The *C*-terminal fragment of CPE (*C*-CPE) has also been shown to effectively target TNFα to ovarian cancer cells [138]. In ovarian cancer, pharmacologic studies have shown a therapeutic advantage to i.p drug therapy and the combination of C-CPE and cytotoxic chemotherapy both i.p may result in enhanced therapeutic effect with reduced systemic toxicity. The fact that ovarian cancer remains confined to the peritoneal cavity for much of its natural history suggests that i.p administration of CPE may provide improved therapeutic responses compared to similar intra-venous doses for those patients with recurrent ovarian cancer [149].

**Figure 2:**
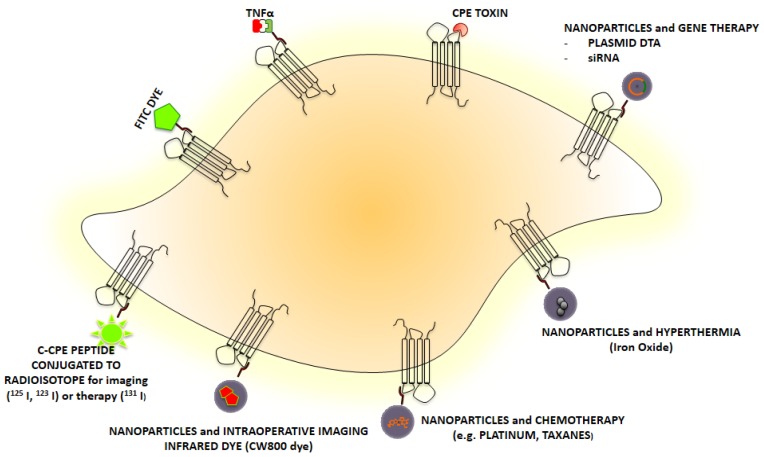
Schematic diagram showing several *C*-CPE based diagnostic and therapeutic approaches including tumor imaging and targeted drug delivery for claudin-3/-4 expressing cells.

As there is a continued need for innovative and effective strategies to treat recurrent/chemo-resistant ovarian cancer, our research group has provided *in vivo* models demonstrating that multiple i.p injections of sublethal doses of CPE every three days significantly inhibited tumor growth in 100% of mice harboring claudin-3 and -4 positive chemotherapy resistant ovarian tumor xenografts [97]. One of our most recent research endeavors in this area was to describe the *in vitro* and *in vivo* bio-activity of the *C*-terminal fragment of CPE as a potential carrier for tumor imaging agents as well as a means of intracellular drug delivery for claudin-3 and -4 positive ovarian neoplasms after i.p injection (Figure 2) [135]. In this study, claudin-3 and -4 expression was determined by qRT-PCR and flow cytometry in several primary ovarian carcinoma cell lines. Both claudin-3 and/or claudin-4 genes were found to be highly expressed in all primary ovarian carcinomas when compared to normal ovarian epithelial cells. The accuracy and specificity of the CPE peptide *in vitro* against primary chemo-resistant ovarian carcinoma cell lines was assessed with cell binding assays, while confocal microscopy and biodistribution assays were performed to evaluate the localization and uptake of FITC-conjugated CPE peptide in the established tumor tissue. Ultimately, this research demonstrated that using FITC-conjugated CPE peptide, there was specific *in vitro* and *in vivo* binding to multiple primary chemo-resistant ovarian carcinoma cell lines. The biodistribution studies in the mice revealed higher uptake of the peptide in tumor cells *versus* normal tissue. A time-dependent internalization of the FITC-conjugated CPE peptide was consistently seen by confocal microscopy in chemotherapy-resistant ovarian carcinoma cells. These findings suggest that CPE peptide is a good candidate as a lead peptide for tumor therapy or for the development of new diagnostic tracers with the possibility of demonstrating disease extent preoperatively or even intra-operatively using near-infrared fluorescent imaging [135].

Further work in the area of chemo-resistant ovarian cancer has demonstrated that CD44+ ovarian cancer stem cells represent a small proportion of cancer cells capable of sustaining tumor growth and chemo-resistance and these cancer stem cells highly express genes encoding claudin-4. Casagrande *et al.* showed that small interfering RNA -mediated knockdown of claudin-3/-4 expression in CD44+ cancer stem cells significantly protected cancer stem cells from CPE-induced cytotoxicity. Here again multiple sublethal doses of i.p CPE proved to be an effective strategy for the eradication of claudin-4 expressing chemo-resistant ovarian cancer stem cells in mice harboring these xenografts with a 100% reduction in tumor burden in 50% of treated mice; *p <* 0.0001 [95]. These studies and others lend support to the efficacy of using recombinant CPE protein in a dose-dependent manner for treating claudin-3 and -4 tumor cells *in vitro* and *in vivo* [97,135]. In general, the *in vivo* application of recombinant CPE did not induce toxin-associated side effects, however repeated administration regionally or loco-regionally was required in order to attain a therapeutic effect [89,150,151]. As progress continues in the molecular understanding of the CPE-claudin interactions, this may potentially lead to the development of enhanced recombinant CPE proteins.

Another novel approach of targeting claudin-3 and -4 expressing ovarian tumor cells is through gene therapy. Intra-tumoral gene transfer of CPE-expressing vectors can be employed for selective suicide gene therapy of claudin-3 and -4 positive tumors and was found to effect a more rapid and effective tumor cell killing *in vitro* and *in vivo* [152]. Cytotoxicity of up to 100% was observed 72 h after gene transfer and was restricted to claudin-3 and -4 expressing tumor lines. Additionally the *in vivo* data from this study revealed significant inhibition of ovarian cancer xenograft growth in SCID mice [152].

## 5. Conclusions

Claudin-3, -4 and -7 are highly expressed in ovarian cancer. While the understanding of the exact role of these proteins in ovarian as well as other human tumors remains poorly defined, substantial experimental evidence has demonstrated an important role for claudin-3 and -4 in ovarian cancer cell invasion and dissemination, resistance to chemotherapy and as target of CPE treatment. In multiple preclinical *in vitro* and *in vivo* models recombinant CPE has been shown to induce a dose-dependent eradication of claudin-3 and -4 tumor cells while the carboxy-terminal fragment of CPE (*i.e.*, CPE_290–319_ binding peptide) has demonstrated promise as a carrier for tumor imaging agents and intracellular delivery of therapeutic drugs [41,151]. The future design and implementation of phase 1 clinical trials in chemo-resistant and recurrent solid tumors will ultimately determine the feasibility and validity of these novel CPE-based theranostic approaches.

## Figures and Tables

**Table 1:** Claudin-1, -3, -4, -5 and -7 expression and function in normal tissues.

Claudin	Function	Tissue specificity	Involvement in disease
Claudin-1	TJ-specific obliteration of the intercellular space through Ca^2+^-independent cell-adhesion activity. Acts as a co-receptor for HCV entry into hepatic cells	Strongly expressed in liver and kidney. Also expressed in heart, brain, spleen, lung and testis	Ichthyosis [61]
Claudin-3	TJ-specific obliteration of the intercellular space through Ca^2+^-independent cell-adhesion activity (CPE is the natural ligand)	Strongly expressed in ovary, lung, pancreas, salivary gland, kidney, adrenal, small intestine, colon and thyroid	Williams-Beuren syndrome [62]
Claudin-4	TJ-specific obliteration of the intercellular space (CPE is the natural ligand)	Strongly expressed in ovary, lung, pancreas, salivary gland, kidney, adrenal, small intestine, colon and thyroid	Williams-Beuren syndrome [62]
Claudin-5	Target molecule of hypoxia	Strongly expressed in vascular endothelial cells. Transiently expressed during development of RPE. Expressed in lung	Velocardiofacial syndrome [63]
Claudin-7	TJ-specific obliteration of the intercellular space. Co-localizes with EPCAM at the lateral cell membrane and TJ	Strongly expressed in kidney, GI tract, thyroid, adrenal gland and lung. Also expressed in prostate tissue	Related to ability of breast cancer cells to disseminate. Downregulation correlates with histological grade [57]

**Table 2:** Claudin expression in gynecologic cancer.

Tumor type	Claudin gene	Expression compared to normal tissues	References
Ovarian	CLDN3	High	[16,18,54,56]
	CLDN4	High	[16,18,43]
	CLDN7	High	[18,84–86]
	CLDN16	High	[17]

Endometrial	CLDN2	High	[87]
	CLDN3	High	[87–89]
	CLDN4	High	[53,88]

Cervical	CLDN1	High	[90,91]
	CLDN2	High	[91]
	CLDN4	High	[91,92]
	CLDN7	High	[90,92]
